# *Salmonella*-Induced Mucosal Lectin RegIIIβ Kills Competing Gut Microbiota

**DOI:** 10.1371/journal.pone.0020749

**Published:** 2011-06-09

**Authors:** Christian Stelter, Rina Käppeli, Claudia König, Alexander Krah, Wolf-Dietrich Hardt, Bärbel Stecher, Dirk Bumann

**Affiliations:** 1 Junior Group, Mucosal Infections, Institute of Immunology, Hannover Medical School, Hannover, Germany; 2 Institute of Microbiology, ETH Zürich, Zürich, Switzerland; 3 Department of Molecular Biology, Max-Planck-Institute for Infection Biology, Berlin, Germany; 4 Arbeitsgruppe Mikrobiota & Infektionen, Max von Pettenkofer-Institute, München, Germany; 5 Infection Biology, Biozentrum, University of Basel, Basel, Switzerland; University of Birmingham, United Kingdom

## Abstract

Intestinal inflammation induces alterations of the gut microbiota and promotes overgrowth of the enteric pathogen *Salmonella enterica* by largely unknown mechanisms. Here, we identified a host factor involved in this process. Specifically, the C-type lectin RegIIIβ is strongly upregulated during mucosal infection and released into the gut lumen. *In vitro*, RegIIIβ kills diverse commensal gut bacteria but not *Salmonella enterica* subspecies I serovar Typhimurium (*S.* Typhimurium). Protection of the pathogen was attributable to its specific cell envelope structure. Co-infection experiments with an avirulent *S.* Typhimurium mutant and a RegIIIβ-sensitive commensal *E. coli* strain demonstrated that feeding of RegIIIβ was sufficient for suppressing commensals in the absence of all other changes inflicted by mucosal disease. These data suggest that RegIIIβ production by the host can promote *S.* Typhimurium infection by eliminating inhibitory gut microbiota.

## Introduction

Diarrhea is an infectious disease that causes high mortality worldwide, especially among children and the elderly [1]. *Salmonella* spp. infection is an important cause of gastroenteritis and has been widely studied because of facile *Salmonella* spp. genetics and excellent animal disease models [2], [3], [4]. Current treatment relies mostly on water and electrolyte supplementation as *Salmonellosis* is often self-limiting in humans [5]. Patients which show septic *Salmonellosis* are treated with antibiotics, but the rise of drug resistant *Salmonella* strains is a current problem in efficient *Salmonella* spp. therapy [5], [6].

The normal intestinal microbiota of mammals counteracts infection by *Salmonella* spp. and other pathogens, a phenomenon referred to as colonization resistance (CR) [7], [8], [9], [10]. The microbiota can impair *Salmonella* spp. by blocking adhesion sites, production of inhibitory or toxic molecules, or stimulation of the host's immune system [11], [12], [13], [14]. However, intestinal inflammation can reduce CR by diminishing inhibitory microbiota and changing its composition. As a result, inflammation promotes high-level *Salmonella enterica* colonization and effective transmission by prolonged fecal shedding [15], [16], [17], [18], [19], [20]. Interestingly, gut inflammation also enhances intestinal infection by other enteropathogenic bacteria such as *Citrobacter rodentium*, *Vibrio cholerae* or *Clostridium difficile* [21], [22], [23].

The mechanism promoting inflammation-mediated overgrowth of enteric pathogens is not fully understood. Inflammation could alter intestinal nutrient availability (i.e. mucus-derived carbohydrates) in a way that increases pathogen growth but diminishes resident microbiota (‘nutrient hypothesis’) [16], [24]. Another reason for *S.* Typhimurium overgrowth in an inflamed gut is the production of tetrathionate from microbiota-derived H_2_S by the inflamed host mucosa [24]. Tetrathionate allows *Salmonella enterica* subspecies I serovar Typhimurium (*S.* Typhimurium) to perform a respiratory metabolism, which confers an advantage in the competition with the microbiota [24]. Alternatively, inflammation induces immune effector mechanisms that kill resident microbiota but not resistant pathogens (‘killing hypothesis’). Indeed, the antimicrobial protein lipocalin-2, which is upregulated in inflamed mice and Rhesus macaques [25], blocks iron uptake in enteric bacteria by sequestering enterochelin. However, *S.* Typhimurium produces salmochelin, a glycosylated form of enterobactin, which is not bound by lipocalin-2 [26], [27]. Hence, *S.* Typhimurium gains a selective advantage in the presence of lipocalin-2 [25]. These data suggest that there is not a single factor leading to *S.* Typhimurium outcompetition of the microbiota but more likely a whole range of parameters playing a role in inflammation.

Antimicrobial peptides (e.g., defensins, cathelicidins) constitute another host defence supporting the mucosal barrier. The C-type lectin RegIIIγ, a member of the Reg gene family, a diverse group of secreted proteins harboring a C-type lectin carbohydrate recognition domain, has been shown to exert antimicrobial effects on commensal bacteria [28]. The closely related RegIIIβ has previously been shown to be upregulated in response to inflammation and infections [29], [30]. Here, we studied the properties and *in vivo* relevance of the C-type lectin RegIIIβ in inflammation-induced microbiota-pathogen competition. This was of interest as RegIII family members are known to affect host-commensal and host-pathogen interactions in the intestine [28], [30], [31]. Our results imply that RegIIIβ is indeed one of the host factors explaining why *S.* Typhimurium can benefit from triggering gut inflammation.

## Materials and Methods

### Ethics statement

All animals were handled in strict accordance with good animal practice and all animal work was approved by local animal care and use committees (license 04/862 Niedersächsisches Landesamt für Verbraucherschutz und Lebensmittelsicherheit; license 201/2007 Kantonales Veterinäramt Zürich).

### Bacterial strains and growth conditions

All *S.* Typhimurium strains used in this study were derivatives of SL1344 *hisG rpsL xyl* [32] (TABLE 1). The virulent and avirulent *S.* Typhimurium strains have been described previously [33], [34] as well as *S.* Typhimurium *ΔaroA* [35]. *Salmonella* Typhimurium mutants with defined gene deletions were obtained using the Lambda phage red recombinase method [36] with primers described at http://falkow.stanford.edu/whatwedo/wanner/ (see also TABLE 2). For comparison of LPS defect mutants, *Salmonella* Typhimurium LT *galE* [37] was used.

**Table 1:** Strains used in this study.

Strain	Genetic information	Strain information	Growth condition	Reference	Figure
*S.* Typhimurium wt	Wild type SL1344	virulent *S.* Typhimurium	LB medium	[33]	Fig. 1, Fig. 2, Fig. S1
*S.* Typhimurium avir	Δ*invG*, *sseD::aphT*	avirulent *S.* Typhimurium	LB medium	[34]	Fig. 1, Fig. 5 Fig. S1
*S.* Typhimurium avir	*invG*::*Cm*, *ssrB::Km*	avirulent *S.* Typhimurium	LB medium	This study	Fig. 4
*S.* Typhimurium Δ*phoP*	*phoP::Km*		LB medium	This study	Fig. 3
*S.* Typhimurium Δ*rfc*	Δ*aroA*, *rfc::Km*		LB medium	This study	Fig. 3
*S.* Typhimurium Δ*rfbP*	Δ*aroA*, *rfbP::Km*		LB medium	This study	Fig. 3
*S.* Typhimurium Δ*galE*	Δ*galE*	LT2	LB medium	[37]	Fig. 3
*S.* Typhimurium Δ*rfaG*	Δ*aroA*, *rfaG::Km*		LB medium	This study	Fig. 3
*S.* Typhimurium Δ*pagL*	Δ*aroA*, *pagL::Km*		LB medium	This study	Fig. 3
*S.* Typhimurium Δ*pagP*	Δ*aroA*, *pagP::Km*		LB medium	This study	Fig. 3
*E. coli* E2		Mouse intestinal isolate, selected for spontaneous Rifampicin resistance	LB medium	This study	Fig. 2, Fig. 3
*E. coli* Nissle 1917		Probiotic *E. coli*	LB medium	[64]	Fig. 3B
*E. coli* CFT073		Uropathogenic *E. coli*	LB medium	[65]	Fig. 3B
*B. subtilis*		168		*Bacillus* Genetic Stock Center	Fig. 3A
*L. reuteri* RR^Rif^		Mouse intestinal isolate, selected for spontaneous Rifampicin resistance	MRS medium, grown anaerobically, 37°C	[15]	Fig. 3B
*L. murinus* RR^Rif^		Mouse intestinal isolate, selected for spontaneous Rifampicin resistance	MRS medium, grown anaerobically, 37°C	This study	Fig. 3B
*E. faecalis*		Mouse intestinal isolate	MRS medium, grown anaerobically, 37°C	This study	Fig. 3B
*C. butyricum* DSM10702		Mouse intestinal isolate	Wilkins Chalgren agar supplemented with 5% defibrillated sheep blood (Oxoid), grown anaerobically	DSMZ, Braunschweig, Germany	Fig. 3B

**Table 2:** Primers used in this study.

Primer	Sequences
*rfbP::Km*	rfbP-P1 (60 nt) 5′-ATT TTA TTT ACA TTA TGC ACG GTC AGA GGG TGA GGA TTA AGT GTA GGC TGG AGC TGC TTC-3′rfbP-P4 (60 nt) 5′-GCT AAT TTA TAC AAT TAT TAT TCA GTA CTT CTC GGT AAG CAT TCC GGG GAT CCG TCG ACC-3′rfbP-F (20 nt) 5′-ACC TGA GTT ACG CTG CTA TG-3′rfbP-R (19 nt) 5′-TCC TGT CAG GTG TGG AAA C-3′
*rfaG::Km*	rfaG-P1 (60 nt) 5′-TGC CGC ATG AGG CAC GCA CCA TAG ATT TGG ACA GCC TGC TGT GTA GGC TGG AGC TGC TTC-3′rfaG-P4 (60 nt) 5′-ATC TTT ACC GCG CCA TAG TGT GGT TAA CGG CGC TTT CAG CAT TCC GGG GAT CCG TCG ACC-3′rfaG-F (19 nt) 5′-CCG GCT GAA GAT GTT ATC G-3′rfaG-R (20 nt) 5′-GCG TCT CCA GCT CTC TGA AC-3′
*pagL::Km*	pagL-P1 (60 nt) 5′-GCC GGT TAA AAA TAA CTA TTG ACA TTG AAA TGG TGG TGG AGT GTA GGC TGG AGC TGC TTC-3′pagL-P4 (60 nt) 5′-TTA CTC CTT CAG CCA GCA ACT CGC TAA TTG TTA TTC AAC TAT TCC GGG GAT CCG TCG ACC-3′pagL-F (20 nt) 5′-TGC TAT ATC AGC CGT TTC TG-3′pagL-R (20 nt) 5′-CTG ATT GGA CAT CTT TCC TG-3′
*pagP::Km*	pagP-P1 (60 nt) 5′-GGT TAA TGT TGT TAT TAT CAC AGT CGA ATT TTT GAA CGG TGT GTA GGC TGG AGC TGC TTC-3′pagP-P4 (60 nt) 5′-TAA GAC TTT TTA ATT CAC AAC TGA AGC ATA CCC TTC CCC AAT TCC GGG GAT CCG TCG ACC-3′pagP-F (20 nt) 5′-ACG CCG TTA ACC CGA TAC TC-3′pagP-R (20 nt) 5′-ACG TCT TTG CTG CCA TCT TC-3′

Non-pathogenic *Escherichia coli* E2 (*stx1− stx2− eaeA− hlyA− espP− katP− astA− recA+ tolC+*) was isolated from BALB/c mice originally obtained from Charles River and maintained at the Hannover Medical School Animal Facility for 12 weeks. Streptomycin- and rifampicin-resistant E2 derivatives were obtained by consecutive selection of spontaneous mutants on media containing rising antibiotic concentrations. *Bacillus subtilis* 168 was obtained from the *Bacillus* Genetic Stock Center. For detailed description of gut commensal strains see TABLE 1.

For mouse infections, bacterial strains were grown for 12 h at 37°C in Luria-Bertani (LB) broth (0.3 M NaCl) and subcultured for 4 h, as described [34].

### Purification of recombinant Proteins

A synthetic sequence optimized for expression of murine RegIIIβ (ProteinID: NP_035166.1) without signal peptide in *E. coli* was constructed (GENEART, Regensburg) and cloned into pET11a (Novagen, Bad Soden). Variant RegIIIβ R135T was constructed using PCR mutagenesis and verified by sequencing. RegIIIβ and the variant were expressed in *E. coli* BL21 (DE3) and purified essentially as described [38]. In brief, cells were induced with 0.1 mM IPTG for 3 h. Cells were harvested by centrifugation, washed with PBS, and frozen at −20°C. Cells were thawed on ice and resuspended in IB buffer (20 mM Tris–HCl, 10 mM EDTA, 1% Triton X-100, 0.1 mg/ml Lysozyme, pH 7.5). Cells were lysed by sonication (7×20 s pulses) and RegIIIβ inclusion bodies were sedimented by centrifugation. The pellet was resuspended in 25 ml IB buffer and sonification/centrifugation was repeated three times to reduce contaminations by intact cells. Purified inclusion bodies were resuspended in denaturing buffer (7 M Guanidine–HCl, 0.15 M reduced glutathione, 2 mM EDTA, 0.1 M Tris–HCl, pH 8.0). Still insoluble material was removed by centrifugation and the supernatant was slowly diluted into ice-cold refolding buffer (0.5 m arginine–HCl, 0.6 mM oxidized glutathione, 50 mM Tris–HCl, pH 8.0). After overnight incubation, insoluble material was removed by centrifugation and the supernatant was concentrated using ultrafiltration with a 10 kDa cut-off membrane (Millipore). The concentrate was dialyzed twice against binding buffer (25 mM MES-NaOH, 25 mM NaCl, pH 6.0). Insoluble material was removed by centrifugation. The final material was homogenous as judged by SDS-PAGE. We verified by omitting lysozyme in the purification protocol that it doesn't contribute to bacterial killing in the *in vitro* assays.

For some experiments, RegIIIβ was trypsinated with 500 ng/ml Trypsin (5000 NF-U mg-1) at a concentration of 50 µg ml^−1^ in binding buffer at 37°C for 2 h. For fluorescence detection, RegIIIβ was covalently linked to Alexa647 (Molecular Probes) following the manufacturer's instructions.

Purification of RegIIIγ has been performed as described previously [38].

### Animal experiments

Mice were purchased from Charles River (Sulzfeld, Germany) or bred at the RCHCI ETH Zurich, Switzerland. Eight to 12 weeks old female BALB/c or C57BL/6 mice were pre-treated with 20 mg streptomycin and 24 h later intragastrically infected with 5×10^7^ CFU of *S.* Typhimurium, as previously described [39].

For some experiments, streptomycin-pretreated MyD88^−/−^ and MyD88^+/−^ mice (C57BL/6 background [40]) were infected with wild-type *S.* Typhimurium and sacrificed 24 h later.

Colonization was determined by plating feces on selective media (MacConkey agar with appropriate antibiotics). In contrast to many non-culturable commensals, the gut luminal cfu data of *S.* Typhimurium (and *E. coli*) obtained by plating always match the cfu data obtained in feces. This is well documented by data from plating, 16S RT PCR, in situ hybridization and immunofluorescence microscopy [9], [15], [39], [60].

To determine the effect of RegIIIβ on bacterial colonization, mice received a single intragastric dose, or daily doses of 80 µg RegIIIβ or 80 µg BSA respectively, in 200 µl binding buffer.

### *In vitro* assays

Binding and cidal activities of RegIIIβ were determined using bacteria from late log liquid cultures. Bacteria were washed and resuspended in binding buffer (25 mM MES-NaOH, 25 mM NaCl, pH 6.0) at a density of 10^6^ CFU ml^−1^. RegIIIβ was added at various concentrations and the mixture was incubated for various times at 37°C. Bacteria were then plated either on LB, Wilkins Chalgren, or MRS agar, and grown under aerobic or anaerobic conditions (7% H2, 10% CO2, 83% N2). As controls ( = 100% survival) Bacteria incubated with binding buffer only, were analyzed.

In some experiments, RegIIIβ was pre-incubated for 10 min with a fivefold (weight/weigth) excess of peptidoglycan (insoluble PGN from *Bacillus subtilis*, Sigma) before addition to bacterial suspensions. Peptidoglycan co-precipitation was determined as described [28].

Bacteria were incubated for 40 min at 4°C with 1 µM fluorescence-labeled RegIIIβ in binding buffer containing 1% bovine serum albumin to minimize unspecific binding. At this low concentration, no cidal effect of RegIIIβ that could potentially affect the results has been detected. Bacteria were washed three times in binding buffer and analyzed using a Calibur flow cytometer (BD Biosciences).

### Antibody generation, WB, and immunohistochemistry

A polyclonal rabbit anti-RegIIIβ antibody was produced by Neosystem (Strasbourg, France) using recombinant RegIIIβ. The antibody was further affinity purified with AminoLink Kit (Thermo Scientific Pierce) using recombinant RegIIIβ. The antibody is specific for RegIIIβ (Fig. S1).

For analysis of intestinal RegIIIβ, cecal contents were resuspended in PBS. Proteins were separated by SDS-PAGE followed by Western blotting using the polyclonal anti-RegIIIβ antibody and chemoluminescent detection (GE Healthcare).

For immunohistochemistry, cecal tissue samples were fixed overnight in PBS with 4% paraformaldehyde at 4°C, incubated in 20% sucrose at 4°C for one day, and snap frozen in O.C.T. compound (Sakura). Cryosections (10 µm) were air dried, permeabilized with 0.1% Triton in PBS, blocked (10% goat serum in PBS), and stained with rabbit anti-RegIIIβ antibody and cy3-labelled anti-rabbit antibody (Jackson). No tissue staining was observed with secondary antibody only. Nuclei were visualized with DAPI. The actin brush border was stained with Phalloidin-Alexa 647 (Fluoprobes). Images were taken with a Axiovert 200 (Carl Zeiss, Inc.) microscope, an Ultraview confocal head (PerkinElmer), and a krypton argon laser (643-RYB-A01; Melles Griot). Infrared, red and green fluorescence was recorded confocally, and blue fluorescence was recorded by epifluorescence microscopy.

### Quantitative RT-PCR

RegIIIβ mRNA levels were quantified using QIAGEN isolation kits, M-MLV reverse Transcriptase RNase H Minus, the QuantiTect SYBR Green PCR kit (Qiagen), and primers as described below. c_t_ values were normalized to GAPDH [41] and represent the median of triplicate analyses compared to non-infected mice. Cycling parameters were 94°C (15 s), 60°C (30 s), 72°C (30 s) in a RotorGene 3000 cycler (Corbett Research, Cambridgeshire, UK). The following Primers were used: GAPDH (5′-GGC TGC CCA GAA CAT CAT CCC TGC AT-3′ and 5′-ACG TCA GAT CCA CGA CGG ACA CAT TGG-3′) and RegIIIβ (5′- CTG CCT TAG ACC GTG CTT TC-3′ and 5′- ATA GGG CAA CTT CAC CTC AC-3′).

### Statistical analysis

Statistical analysis was performed using the Student T-Test except for long-term colonization where the Mann Whitney U-test was applied. P ≤ 0.05 was considered to be statistically significant.

## Results

### *S.* Typhimurium infection induces intestinal RegIIIβ expression

**Figure 1:**
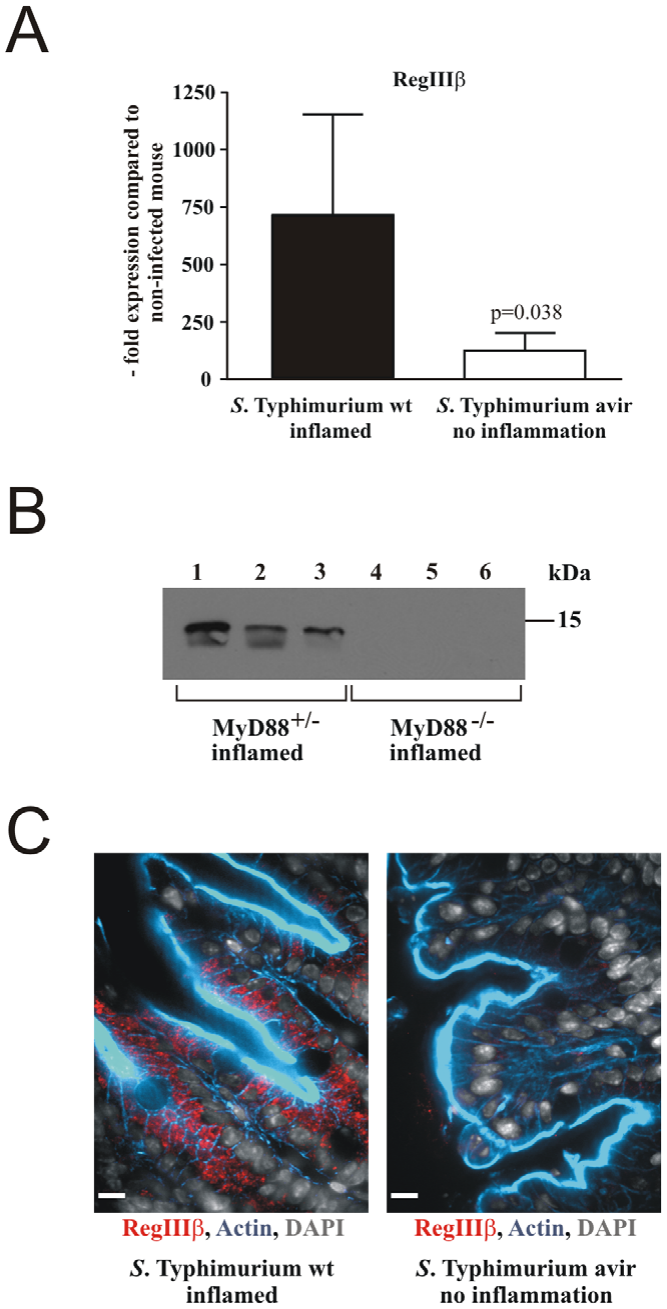
*S.* Typhimurium-induced RegIIIβ expression in mouse intestine. A) RegIIIβ expression was analyzed in the cecum of streptomycin-treated C57BL/6 mice infected for 24 h with *S.* Typhimurium wt and *S.* Typhimurium avir, respectively. Expression was analyzed by quantitative real time PCR normalized to GAPDH mRNA levels (fold expression versus unmanipulated mouse). B) Western Blot of intestinal contents obtained from *S.* Typhimurium infected MyD88^+/−^ and MyD88^−/−^ litter mates 24 h post infection using a polyclonal antibody to RegIIIβ. Samples 1–3 were obtained from infected MyD88^+/−^ mice and samples 4–6 from MyD88^−/−^ litter mates. C) Immunohistochemistry of the cecal mucosa of *S.* Typhimurium infected mice 24 h post infection (grey, nuclei (DAPI); blue, actin (phalloidin); red, RegIIIβ). Scale bar: 10 µm.

The C-type lectin RegIIIβ is highly induced in rat intestinal tissues during enteric *Salmonellosis* [42], [43]. We aimed at analyzing the role of RegIIIβ in microbiota-pathogen competition in the streptomycin-treated mouse model for *S.* Typhimurium induced gut inflammation. To initially confirm validity of previous data for this model, we performed quantitative real time PCR of RegIIIβ mRNA on cecal tissue (Fig. 1A). Mice infected with an avirulent *S.* Typhimurium strain that fails to cause intestinal inflammation, show slightly upregulated RegIIIβ mRNA levels compared to baseline levels in non-infected control mice. In contrast, mice infected with wild-type *S.* Typhimurium (wt) show drastically increased levels (∼10-fold; Fig. 1A).

To confirm qPCR data on protein level, we analyzed intestinal contents in Western blots using an affinity-purified polyclonal antibody to RegIIIβ (Fig. S2A). This antibody recognizes a major 15 kDa band and a weaker band of approx. 14 kDa, which likely correspond to RegIIIβ and a proteolytically processed form (Fig. 1B). Proteolytic cleavage by trypsin has been reported for the closely related C-type lectin RegIIIγ [28]. As trypsin is present in intestinal contents, RegIIIβ processing is likely to occur *in vivo*.

Quantification using purified recombinant RegIIIβ as a reference indicated that RegIIIβ was present at baseline levels of less than 1 µg/g intestinal contents in uninfected mice, but increased to levels of some 350 µg/g in mice infected with virulent *S.* Typhimurium (Fig. S2B).

*S.* Typhimurium-induced RegIIIβ upregulation critically depended on the innate immunity adapter protein MyD88 (Fig. 1B) as previously reported in case of RegIIIβ induction by the commensal microbiota [44]. The same is true for the closely related C-type lectin RegIIIγ in a *L. monocytogenes* infection model [30]. Immunohistochemistry of intestinal tissue sections revealed weak RegIIIβ levels in *S.* Typhimurium infected mice, which were non-inflamed, but prominent RegIIIβ expression in intestinal epithelial cells in mice with gut inflammation (Fig. 1C). These data confirmed and extended previous observations of RegIIIβ upregulation in intestinal tissues in response to virulent *Salmonella* spp..

### RegIIIβ kills commensal bacteria but not *S.* Typhimurium

**Figure 2:**
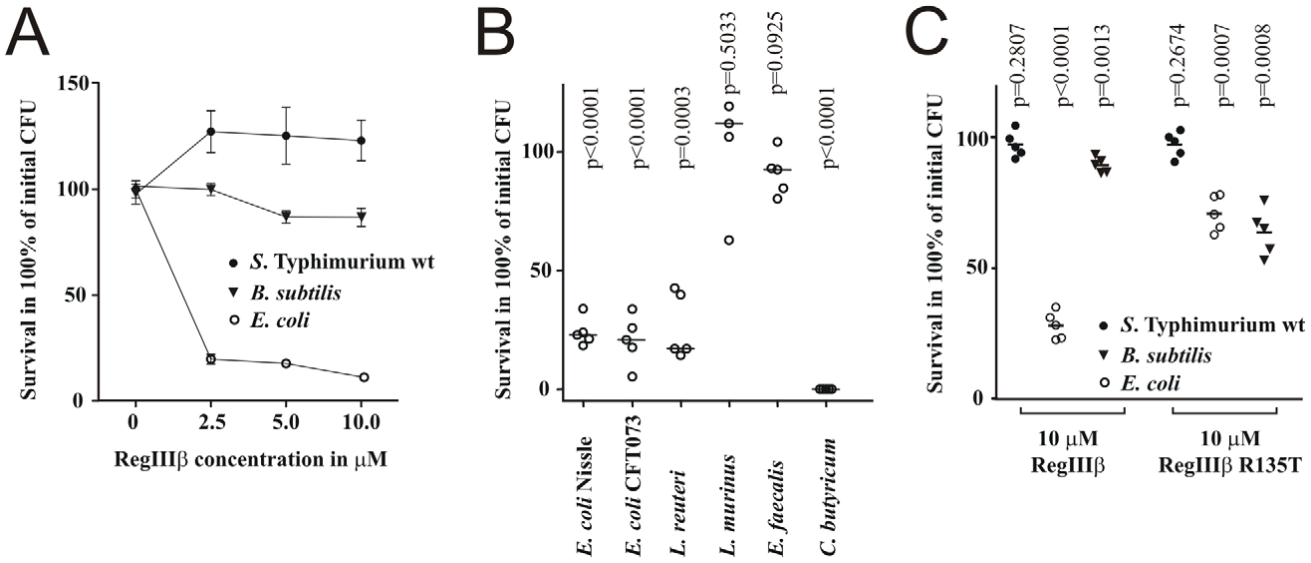
RegIIIβ has bactericidal activity against several Gram^+^ and Gram^−^ intestinal bacteria. A) Survival of *S.* Typhimurium (filled circles), *E. coli* (open circles), and *Bacillus subtilis* (triangles) after 30 min incubation with various concentrations of RegIIIβ. Means ± SD of five independent replicates are shown. B) Survival of various intestinal commensal bacteria after incubation with 10 µM RegIIIβ (1, *E. coli* Nissle; 2, *E. coli* CFT073; 3, *Lactobacillus reuteri*; 4, *Lactobacillus murinus*; 5, *Enterococcus faecalis*; 6, *Clostridium butyricum*). C) Bactericidal activities of 10 µM RegIIIβ and 10 µM RegIIIβ R135T with altered putative binding site against *S.* Typhimurium, *E. coli* and *B. subtilis* (symbols as in A). Statistical significances for deviation from results obtained for bacteria incubated with buffer only are shown.

The C-type lectin RegIIIγ has been reported to have bactericidal effects. To monitor the antimicrobial activity of closely related RegIIIβ on different bacterial species, we produced recombinant RegIIIβ. We tested its antibacterial spectrum in *in vitro* killing assays against *S.* Typhimurium wt, *E. coli* and *B. subtilis*. *B. subtilis* and *E. coli* were chosen as these strains were used in other studies assessing bacterial susceptibility to RegIIIγ [28]. Since *Bacillus* spp. is not a normal member of the GI tract, several other Gram**^+^** bacteria typical for the GI tract were also included in the analysis (*Lactobacillus* spp., *Enterococcus* spp., *Clostridium* spp.). Aliquots of bacterial suspensions were incubated with recombinant RegIIIβ and bacterial survival was analyzed after 30 minutes. The cfu of the negative control (no RegIIIβ) was taken as 100% survival rate. Interestingly, recombinant purified RegIIIβ had bactericidal activity against *E. coli* but not *S.* Typhimurium and *B. subtilis*. We tested an extended panel of commensal gut bacteria but found no clear killing preference for Gram**^+^** vs. Gram**^−^** species (Fig. 2A, B). The related intestinal C-type lectin, RegIIIγ is known to selectively kill Gram**^+^** bacteria [28]. These data suggested that the two lectins may have complementary antibacterial profiles. Effective doses for bactericidal activity were below 2.5 µM (Fig. 2A, Fig. S2B), which is in the range of the RegIIIβ concentrations observed in the *S.* Typhimurium-infected intestine (see above). Since *S.* Typhimurium was found to be resistant to RegIIIβ, we hypothesized that *S.* Typhimurium-induced RegIIIβ could thus affect some commensal microorganisms in the intestinal lumen upon gut inflammation, and thereby contribute to the substantial ecological changes in gut microbiota and observed pathogen overgrowth during *S.* Typhimurium wt infection [15], [19], [45].

### Molecular structure correlates with bactericidal activity

To identify the mechanisms of *S.* Typhimurium resistance to RegIIIβ we decided to determine the molecular target of RegIIIβ on the bacterial cell envelope. An excess of peptidoglycan (PGN) completely blocked RegIIIβ bactericidal activity (data not shown) consistent with the previously reported role of PGN as RegIIIβ target [46]. Although RegIIIβ did bind to bacteria containing various PGN types, including *S.* Typhimurium *in vivo* (data not shown), bactericidal activity was preferentially directed against particular PGN types. Specifically, some sensitive bacteria carried the negatively charged amino acid residue diaminopimelic acid at position 3 of the PGN pentapeptide (PGN-DAP: *E. coli*, *Clostridium butyricum*), whereas most resistant bacteria carried a neutral or positively charged amino acid at this position (*B. subtilis*, amidated diaminopimelic acid; *Lactobacillus casei*, *L. murinus* and *Enterococcus faecalis*, lysine) [47], [48], [49]. Interestingly, loop 2 of RegIIIβ, which is homologous to the usual C-type lectin binding site but apparently not involved in binding the carbohydrate portion of peptidoglycan [46], contains a positively charged arginine (R135), while the equivalent residue is a threonine in RegIIIγ which kills bacteria with PGN-Lys. In an unrelated insect peptidoglycan binding protein PGRP-LE a similar arginine - threonine substitution controls selective activity against PGN-DAP *vs.* PGN-LYS [50]. To test the hypothesis that arginine 135 of RegIIIβ promotes selective cidal interaction with negatively charged PGN, we generated a RegIIIβ R135T variant by site-directed mutagenesis. RegIIIβ R135T had weaker cidal activity against *E. coli* but gained cidal activity against *B. subtilis* that was lacking in the wild-type protein (Fig. 2C) supporting the role of amino acid 135 in selective interaction with certain PGN types. The still moderate activity of RegIIIβ R135T against *B. subtilis* suggested multiple additional interactions as observed for other PGN-binding proteins [51]. This was also evident from wild-type RegIIIβ resistance of *Lactobacillus reuteri* despite its Lys-type peptidoglycan.

### The O-antigen protects *S.* Typhimurium against RegIIIβ killing

**Figure 3:**
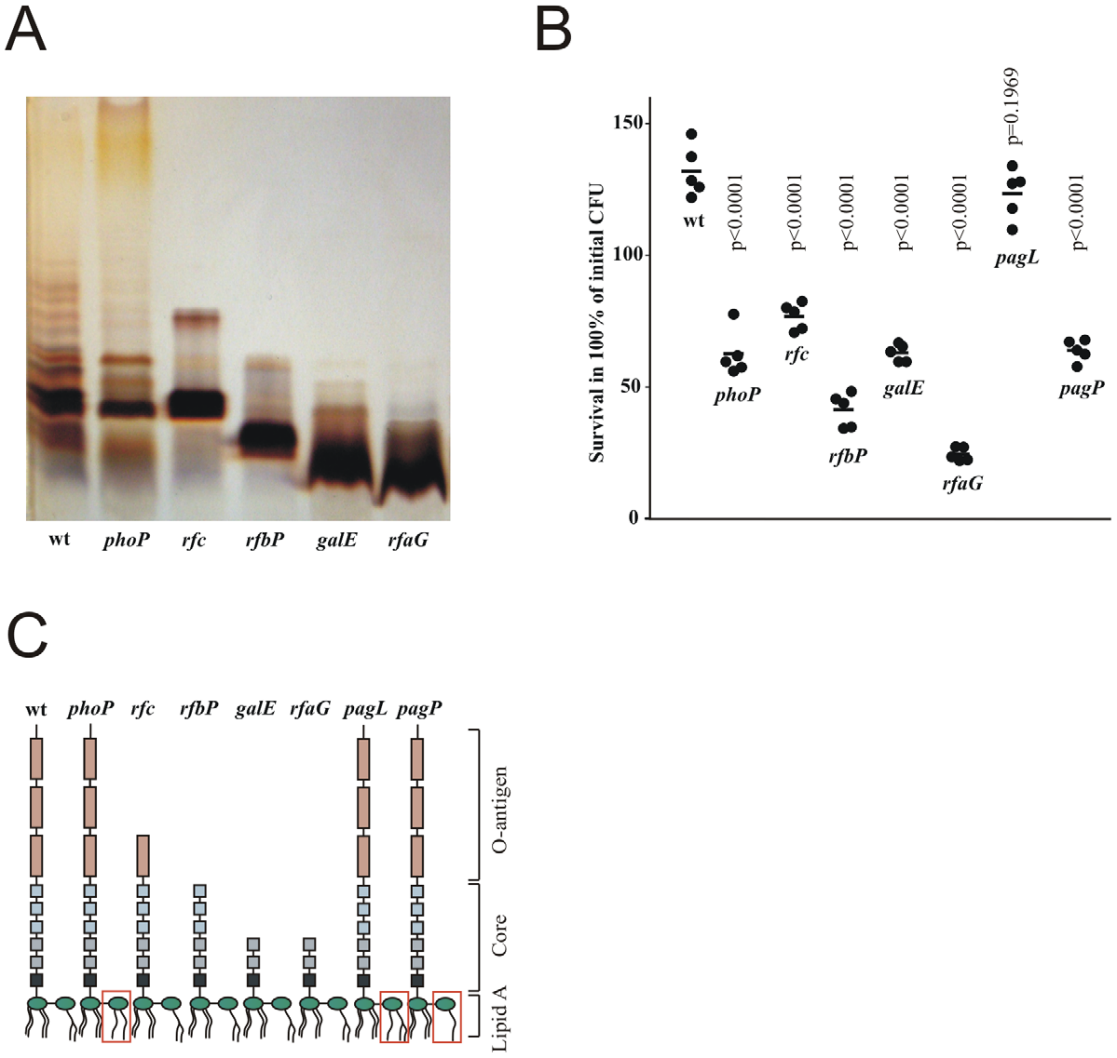
The O-antigen mediates *S.* Typhimurium resistance against RegIIIβ. A) SDS-PAGE (silver stain) of lipopolysaccharide of wild-type *S.* Typhimurium (lane 1) and mutants (*phoP*, *rfc*, *rfbP*, *galE*, *rfaG*). B) Bactericidal activity of 10 µM RegIIIβ against various *S.* Typhimurium mutants. Statistical significances for deviation from results obtained for wildtype bacteria are shown. C) Schematic representation of *Salmonella* spp. LPS-forms.

RegIIIβ did not kill PGN-DAP containing *S.* Typhimurium but did kill *E. coli* containing the same type of PGN (Fig. 2A). This observation was consistent with the uncompromised viability of *Salmonella enterica* isolated from infected gut contents [52]. As one possible resistance mechanism, *Salmonella* spp. outer membrane lipopolysaccharides (LPS) [53] could restrict access of RegIIIβ to its target peptidoglycan in the periplasm. To test this hypothesis, we compared isogenic *S.* Typhimurium mutants with defined defects in LPS biosynthesis. These mutants lacked the O-antigen polymerase (rfc), the complete O-antigen (rfbP), the outer core and the O-antigen (galE) and the inner core, outer core and the O-antigen (rfaG) and certain lipid-A modifications (phoP, pagL and pagP) (Fig. 3C). Shortening of the polysaccharide component of LPS (Fig. 3A) progressively increased *S.* Typhimurium susceptibility to RegIIIβ-mediated killing (Fig. 3B) as observed for other antimicrobial molecules [54]. Moreover, PhoP-dependent expression of *pagP* (involved in hepta-acetylation of lipid A with palmitate [55]) but not *pagL* (catalyzes the 3-O-deacylation of lipid A at position 3 [56]) was essential for *S.* Typhimurium resistance to RegIIIβ (Fig. 3B). These data suggested that in wild-type *S.* Typhimurium carrying a rigid LPS layer, RegIIIβ had limited access to peptidoglycan and therefore weak anti - *S.* Typhimurium activity. Interestingly, *E. coli* has similar LPS modification capabilities, but under our experimental *in vitro* conditions, these mechanisms were apparently insufficient to mediate resistance against RegIIIβ in various *E. coli* strains.

### RegIIIβ selectively suppresses *E. coli* in a simple co-colonization model

The *in vitro* data suggested that RegIIIβ killed various bacterial species but not *S.* Typhimurium. *S.* Typhimurium-induced RegIIIβ could thus contribute to ecological changes in gut microbiota during *S.* Typhimurium infection [15], [19], [45]. To test this hypothesis, we established a simple experimental mouse model employing intestinal colonization of streptomycin-pretreated mice [39] with *S.* Typhimurium and a molecularly defined (see Material & Methods sections) non-pathogenic, streptomycin-resistant *Escherichia coli* strain (*E. coli* E2) from our mouse colony. *S.* Typhimurium colonization preferentially occurs in individuals with high initial *E. coli* abundance [9], which suggests potential relevance for *S.* Typhimurium/*E. coli* competition.

**Figure 4:**
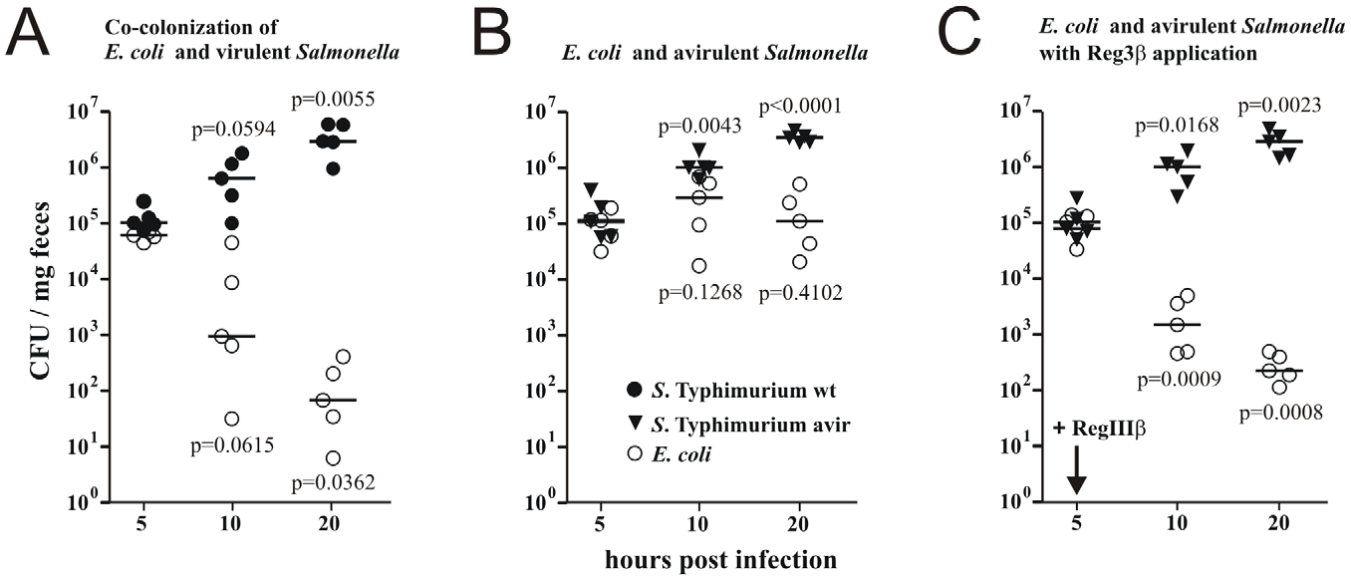
RegIIIβ is sufficient to suppress *E. coli* during competitive growth in the non-inflamed gut. A) Colonization of streptomycin-pretreated mice with virulent *S.* Typhimurium wt (filled circles) and non-pathogenic *E. coli* E2 (open circles). Virulent *S.* Typhimurium induces gut inflammation and suppresses co-colonization with *E. coli*. B) Avirulent *S.* Typhimurium permits *E. coli* co-colonization. C) A single dose of RegIIIβ is sufficient to complement the suppression defect of avirulent *S.* Typhimurium. Each data point represents one mouse. Statistical significances for deviation from results obtained at 5 h are shown.

When given individually, both *E. coli* E2 and *S.* Typhimurium colonized the mouse intestine at high levels in streptomycin-treated mice (data not shown). Virulent *S.* Typhimurium induced intestinal inflammation as previously reported, while an avirulent *S.* Typhimurium *invG ssrB* strain as well as the *E. coli* E2 isolate did not induce obvious pathology (data not shown) in agreement with previous reports [15], [39], [57], [58], [59]. Interestingly, virulent *S.* Typhimurium wt suppressed co-administered *E. coli* E2 (Fig. 4A), while avirulent *S.* Typhimurium permitted stable high level co-colonization of both species (Fig. 4B). Our simple co-colonization model thus reproduced important aspects of *S.* Typhimurium-induced commensal suppression.

To test whether the host factor RegIIIβ might be sufficient for *E. coli* E2 *in vivo* suppression, we administered recombinant RegIIIβ to mice co-colonized by *E. coli* E2 and avirulent *S.* Typhimurium. A single dose of 80 µg RegIIIβ (similar to endogenous RegIIIβ levels induced by virulent *S.* Typhimurium wt, see above) administered at 5 h post infection rapidly suppressed *E. coli* but did not affect *S.* Typhimurium gut colonization (Fig. 4C). RegIIIβ was thus sufficient for suppressing commensal *E. coli* E2 even in the absence of all other mechanisms that might further enhance this effect in the inflamed gut.

### RegIIIβ supports *S.* Typhimurium infection in the presence of complex gut microbiota

A complex gut microbiota strongly inhibits *S.* Typhimurium gut colonization [9], [15]. Through induction of RegIIIβ (Fig. 1A), *S.* Typhimurium might exploit the host to suppress competing microbiota. To test this hypothesis, the simple *E. coli*/*S.* Typhimurium co-colonization model in streptomycin-pretreated mice was inappropriate. The competing microbiota is already weakened in this model. Consequently, even avirulent *S.* Typhimurium can efficiently colonize for several days even in the absence of intestinal inflammation.

**Figure 5:**
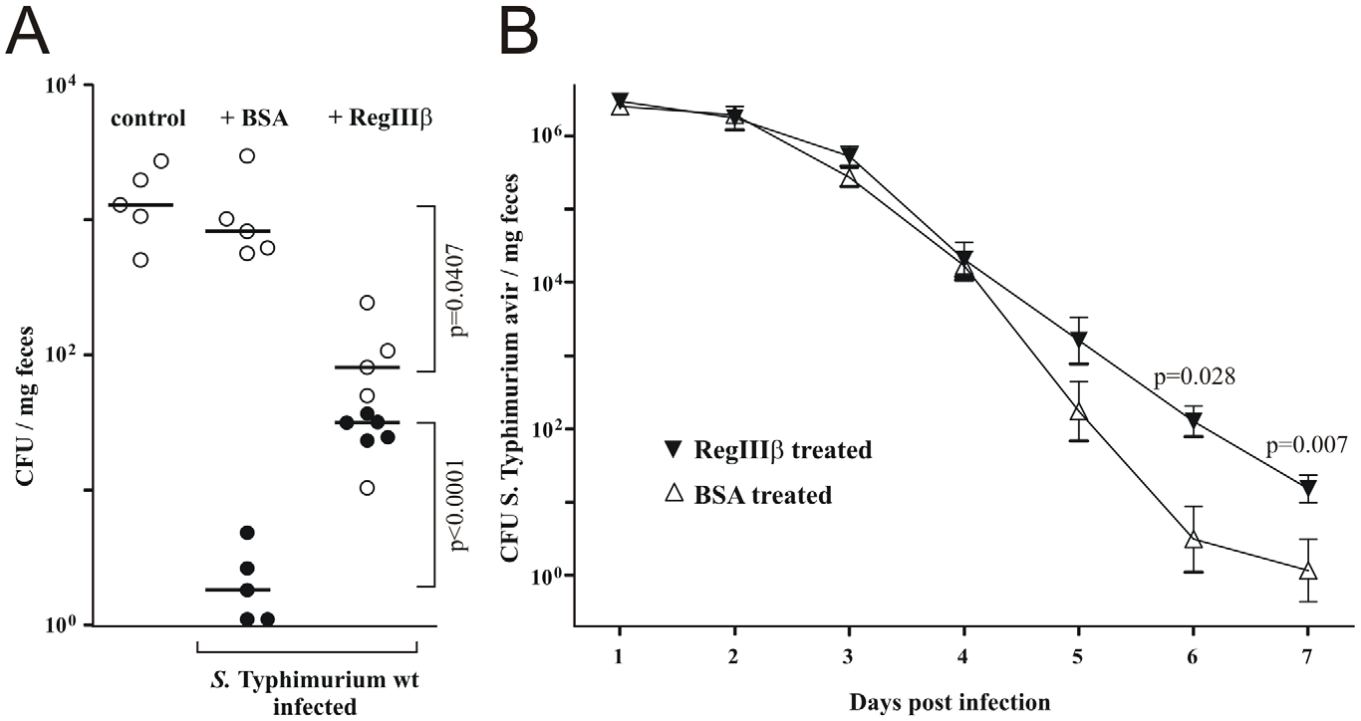
RegIIIβ facilitates *S.* Typhimurium colonization in mice with complex microbiota. A) Colonization of mice with normal intestinal microbiota (no streptomycin pre-treatment) with facultative aerobes (open circles, colonies on MacConkey agar plates; left). Colonization levels of facultative aerobes after a single oral dose of BSA or RegIIIβ and subsequent infection with *S.* Typhimurium (closed circles; middle and right). A single dose of RegIIIβ suppressed resident facultative aerobic bacteria and partially relieved colonization resistance against *S.* Typhimurium. Statistical significances for deviation from results obtained with BSA alone are shown. B) RegIIIβ facilitates prolonged gut colonization by avirulent *S.* Typhimurium in streptomycin-pretreated mice. Daily RegIIIβ doses (80 µg in 200 µl) prolonged *S.* Typhimurium colonization (filled down triangles) compared to control mice that received bovine serum albumin (open up triangles). In panel A) one data point represents one mouse; in panel B) one data point represents the median with SD of 5 mice. Statistical significances for deviation from results obtained for BSA alone are shown. Comparable data were obtained in an independent experiment.

To test if RegIIIβ-induction could offer *S.* Typhimurium any benefit under more natural conditions, we tested conventional mice containing a normal gut microbiota (no streptomycin pretreatment). We determined the fecal density of facultative aerobic bacteria in our mouse colony by plating (Fig. 5A). This technique closely reflects the gut luminal bacterial counts based on previous data from plating, 16S RT PCR, in situ hybridization and immunofluorescence microscopy [9], [15], [39], [60]. However, it is important to notice that luminal (or fecal) counts do not necessarily correlate with mucosal colonization levels. One group of mice was orally treated with a single dose of BSA, the other group with recombinant RegIIIβ, both followed by an oral dose of *S.* Typhimurium (5×10^7^ cfu, by gavage). BSA-treated mice did not show efficient *S.* Typhimurium luminal colonization (Fig. 5A) as observed previously [9], [39], [61]. However, oral treatment with a single dose of RegIIIβ suppressed endogenous Gram**^−^** facultative aerobic microbiota and enabled subsequent *S.* Typhimurium luminal colonization at moderate levels (Fig. 5A).

In another model, we followed long-term shedding of *S.* Typhimurium in streptomycin-pretreated mice during recolonization with competing microbiota, which terminates pathogen growth in the gut after approximately 4 days, in the absence of mucosal inflammation [15], [60]. Due to its defect to trigger inflammation, the avirulent *S.* Typhimurium strain is overgrown by the competing commensal flora. We aimed at testing if oral supplementation of RegIIIβ could interfere with reemerging microbiota-induced colonization resistance. Indeed, daily RegIIIβ administration significantly prolonged *S.* Typhimurium fecal shedding (Fig. 5B). The combined data showed that RegIIIβ can enhance *S.* Typhimurium gut luminal colonization in the presence of complex commensal microbiota.

## Discussion

*S.* Typhimurium induces a complex inflammatory response in the intestinal mucosa. This inflammation has a profound impact on gut microbiota composition. Among others, microbiota that competes with *S.* Typhimurium is suppressed whereas *S.* Typhimurium colonization is promoted. The molecular mechanisms of this process are probably complex and poorly understood [10]. On one side, altered nutrient availability in the inflamed gut may lead to positive selection of the pathogen over the commensal flora [16], [24]. On the other side numerous host defense factors are differentially regulated during intestinal inflammation [25], [42], [43], [62] and could mediate selective killing of the commensal microbiota [17], [20]. Their relevance for facilitating *S.* Typhimurium colonization has remained unclear.

Among the host factors induced by *S.* Typhimurium, the C-type lectin RegIIIβ is particularly strongly upregulated. RegIIIβ can block mucosal infections with pathogenic *Yersinia* spp. although it does not inhibit *Yersinia* spp. directly [31]. RegIIIβ is also highly related to another C-type lectin, RegIIIγ that kills Gram**^+^** bacteria [28] and protects against mucosal *Listeria* infections [30]. For these reasons, we studied the role of RegIIIβ in enteric salmonellosis.

We showed that *S.* Typhimurium infection induced elevated RegIIIβ protein levels in the intestine. Recombinant RegIIIβ bound peptidoglycan *in vitro* and killed a diverse set of both Gram**^−^** and Gram**^+^** bacteria but not *S.* Typhimurium. Site-directed mutagenesis of RegIIIβ and comparison of various *S.* Typhimurium mutants suggested that peptidoglycan structure and lipopolysaccharide composition (for Gram**^−^** bacteria) might explain the bactericidal activity spectrum of RegIIIβ. Recently published work points out that HIP/PAP (hepatointestinal pancreatic/pancreatitis associated protein), a human ortholog of RegIIIγ interacts with the carbohydrate moiety of peptidoglycan which may be also the case for RegIIIβ [46]. The observed antibacterial spectrum of RegIIIβ was clearly different from the reported spectrum of the closely related RegIIIγ suggesting that these two mucosal lectins might serve complementary functions in modulation of gut microbiota. We hypothesize that *S.* Typhimurium is protected against RegIIIβ dependent killing by specific modifications mediating a rigid LPS layer. Possibly, differences between *S.* Typhimurium and *E. coli* in PhoP-dependent regulatory circuits [63] and/or subtle differences in LPS structure might affect the susceptibility for RegIIIβ. Future work will have to address the structural mechanisms underlying differential RegIIIβ killing of *E. coli* and *S.* Typhimurium.

Doses in the range of endogenous RegIIIβ levels were orally administered and could efficiently suppress *E. coli* gut colonization and facilitate *S.* Typhimurium colonization. Our findings suggest that RegIIIβ may contribute to inflammation-induced suppression of competing microbiota although we have no direct evidence of RegIIIβ playing a role *in vivo*. Experiments using RegIIIβ ko mice may aid clarifying this issue.

Presently, we do not know the exact effects of RegIIIβ treatment on microbiota composition and function. This might be an important topic for future research. However, effects resulting from RegIIIβ feeding were moderate compared to the much stronger facilitation of *S.* Typhimurium colonization in mice with acute intestinal inflammation. Thus, additional host factors and bacterial fitness factors apparently also contribute significantly to inflammation-induced microbiota changes and pathogen overgrowth. Possible candidates for these additional factors include the paralogue RegIIIγ with a complementary bactericidal spectrum, defensins and other yet unidentified, *Salmonella*-encoded fitness factors. Further work will be required to elucidate the respective relevance and the unknown mechanisms, and how they might be integrated with the function of the previously identified host and bacterial factors in the microbiota-pathogen interplay in the inflamed intestine.

## Supporting Information

Figure S1 **RegIIIβ levels in intestinal content of C57BL/6 mice.** A) Western Blot of mouse intestinal contents with a purified polyclonal antiserum to RegIIIβ. Samples 1–3 were obtained from streptomycin-treated mice infected with *S.* Typhimurium avir whereas samples 4–6 were taken from mice infected with virulent *S.* Typhimurium 1 day post infection. B) Western Blot showing different dilutions of recombinant RegIIIβ compared to intestinal content (lane 6) of mouse infected with virulent *S.* Typhimurium. (lane 1: 0.552 µg; lane 2: 0.276 µg; lane 3: 0.138 µg; lane 4: 0.069 µg; lane 5: 0.0345 µg; lane 6: 0.26 mg of intestinal content). Lane 6 is equivalent to 92 ng of RegIIIβ (analysis via AlphaImager3400) which results in ∼350 µg/g RegIIIβ.
(TIF)

Figure S2 **Specificity of purified rabbit polyclonal RegIIIβ antibody.** Western Blot with RegIIIβ purified antiserum on same amounts of recombinant RegIIIβ and recombinant RegIIIγ.
(TIF)
