# Supplementary figures and images for: Salmonella-Induced Mucosal Lectin RegIIIβ Kills Competing Gut Microbiota
Source: PLoS One. 2011 Jun 9;6(6):e20749. doi: 10.1371/journal.pone.0020749 (PMC3111430; doi:10.1371/journal.pone.0020749)

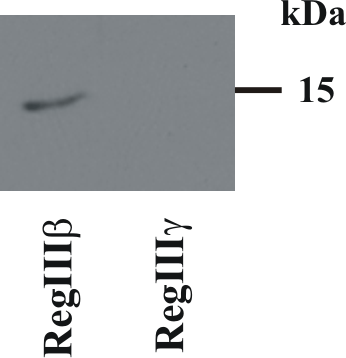

Supplement: Figure S1 — RegIIIβ levels in intestinal content of C57BL/6 mice. A) Western Blot of mouse intestinal contents with a purified polyclonal antiserum to RegIIIβ. Samples 1–3 were obtained from streptomycin-treated mice infected with S. Typhimurium avir whereas samples 4–6 were taken from mice infected with virulent S. Typhimurium 1 day post infection. B) Western Blot showing different dilutions of recombinant RegIIIβ compared to intestinal content (lane 6) of mouse infected with virulent S. Typhimurium. (lane 1: 0.552 µg; lane 2: 0.276 µg; lane 3: 0.138 µg; lane 4: 0.069 µg; lane 5: 0.0345 µg; lane 6: 0.26 mg of intestinal content). Lane 6 is equivalent to 92 ng of RegIIIβ (analysis via AlphaImager3400) which results in ∼350 µg/g RegIIIβ. (TIF) [file pone.0020749.s001.tif]

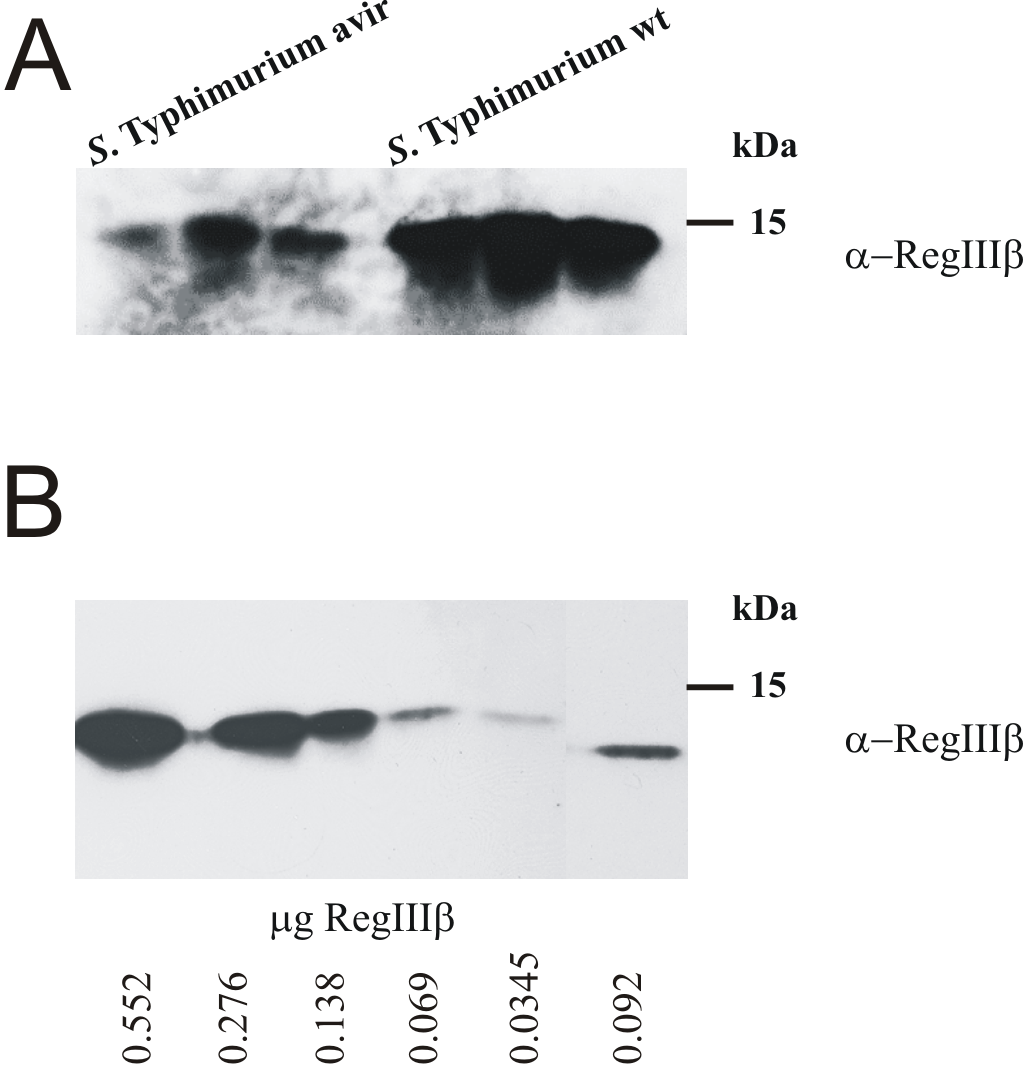

Supplement: Figure S2 — Specificity of purified rabbit polyclonal RegIIIβ antibody. Western Blot with RegIIIβ purified antiserum on same amounts of recombinant RegIIIβ and recombinant RegIIIγ. (TIF) [file pone.0020749.s002.tif]
